# Loss of p16^INK4A^ stimulates aberrant mitochondrial biogenesis through a CDK4/Rb-independent pathway

**DOI:** 10.18632/oncotarget.19862

**Published:** 2017-08-03

**Authors:** Ethika Tyagi, Bin Liu, Chelsea Li, Tong Liu, Jared Rutter, Douglas Grossman

**Affiliations:** ^1^ The Huntsman Cancer Institute, University of Utah Health Sciences Center, Salt Lake, Utah, USA; ^2^ The Department of Dermatology, University of Utah Health Sciences Center, Salt Lake, Utah, USA; ^3^ The Department of Biochemistry, University of Utah Health Sciences Center, Salt Lake, Utah, USA; ^4^ The Howard Hughes Medical Institute, University of Utah Health Sciences Center, Salt Lake, Utah, USA

**Keywords:** p16, mitochondria, CDK4, migration, fibroblast

## Abstract

The tumor suppressor p16INK4A (p16) inhibits cell cycle progression through the CDK4/Rb pathway. We have previously shown that p16 regulates cellular oxidative stress, independent of its role in cell cycle control. We investigated whether loss of p16 had a direct impact on the mitochondria. We found that p16-null primary mouse fibroblasts (PMFs) displayed increased mitochondrial mass and expression of mitochondrial respiratory subunit proteins compared to wild-type (WT) PMFs. These findings in p16-null PMFs were associated with increased expression of the mitochondrial biogenesis transcription factors PRC and TFAM. On the other hand, p16-deficient PMFs demonstrated reduced mitochondrial respiration capacity consistent with electron microscopy findings showing that mitochondria in p16-deficient PMFs have abnormal morphology. Consistent with increased mitochondrial mass and reduced respiratory capacity, p16-deficient PMFs generated increased mitochondrial superoxide. One biological consequence of elevated ROS in p16-deficient PMFs was enhanced migration, which was reduced by the ROS scavenger N-acetylcysteine. Finally, p16-deficient PMFs displayed increased mitochondrial membrane potential, which was also required for their enhanced migration. The mitochondrial and migration phenotype was restored in p16-deficient PMFs by forced expression of p16. Similarly, over-expression of p16 in human melanocytes and A375 melanoma cells led to decreased expression of some mitochondrial respiratory proteins, enhanced respiration, and decreased migration. Inhibition of Rb phosphorylation in melanocytes and melanoma cells, either by addition of chemical CDK4 inhibitors or RNAi-mediated knockdown of CDK4, did not mimic the effects of p16 loss. These results suggest that p16 regulates mitochondrial biogenesis and function, which is independent of the canonical CDK4/Rb pathway.

## INTRODUCTION

The *CDKN2A* locus is among the most common sites of genetic alteration in human cancers [1]. The p16^CDKN2A^ protein (hereafter referred to as p16) binds cyclin-dependent kinases (CDK) to prevent their phosphorylation of the retinoblastoma (Rb) protein which sequesters E2F transcription factors that control transcription of S-phase genes [2]. Loss of p16 function may promote cellular proliferation and impair cell cycle arrest or senescence, allowing survival of genetically damaged cells [3]. Individuals with germline mutations in p16 are predisposed to melanoma [4, 5]. Although p16 may interact with both CDK4 and CDK6, p16 inhibition of CDK4 may be more important than CDK6 given that some melanoma-prone families have inherited activating mutations in CDK4, while none with activating CDK6 mutations have been described [6]. We initially reported that p16 controls levels of reactive oxygen species (ROS), independent of its cell cycle regulatory function [7, 8]. These findings led us to propose that this oxidative regulatory function of p16 represents an alternative tumor suppressor function, although the mechanism(s) through which p16 regulates oxidative stress were unclear.

Mitochondria are the primary source of usable energy, biosynthetic intermediates and metabolic regulation in cells, and their function remains essential for cancer cells [9]. Cancer growth may be supported by increased mitochondrial biogenesis and respiratory capacity [10, 11], which are controlled by the master transcriptional cofactors peroxisome proliferator-activated receptor γ coactivator (PGC-1) α and β or the less studied PGC-1-related coactivator PRC [12, 13]. Mutations in mitochondrial DNA and in nuclear genes encoding mitochondrial proteins have been found in several human tumor types [14–16] and mitochondrial dysfunction may promote cancer development by enhancing ROS production and tumor cell migration and invasion [17–19]. Here, we report that loss of p16 in both primary untransformed cells and melanoma cells causes aberrant mitochondrial biogenesis associated with increased mitochondrial mass and membrane potential but impaired respiration. This phenotype can be rescued by p16 over-expression, but not by knockdown or chemical inhibition of CDK4, suggesting that mitochondrial regulation by p16 is driven by a CDK4/Rb-independent pathway. We hypothesize that this alternative tumor suppressor function of p16 may be important in cancers where mitochondrial dysfunction and ROS generation promotes tumor development and metastasis.

## RESULTS

### p16-deficient cells have greater mitochondrial mass and elevated expression of respiratory complex and mitochondrial coactivator proteins

**Figure 1:**
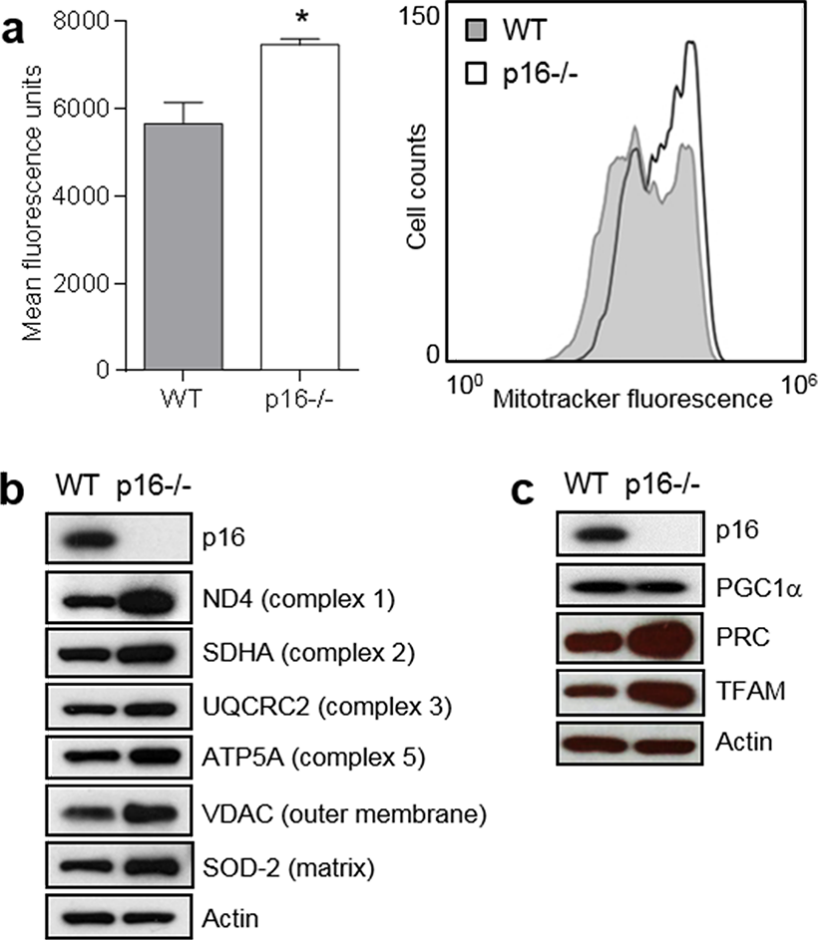
p16-deficient cells display increased mitochondrial mass and biogenesis **a.** Mitochondrial mass. Left, wild-type (WT) and p16^−/−^ primary fibroblasts were stained with MitoTracker-Green, and analyzed by flow cytometry. Error bars indicate SEM from triplicate determinations, *P = 0.02. Right, representative histogram. **b.** Western blotting for mitochondrial structural proteins in WT and p16^−/−^ fibroblasts, with Actin as a loading control. **c.** Western blotting for proteins in WT and p16^−/−^ fibroblasts that regulate mitochondrial biogenesis, with Actin as a loading control.

The association of mitochondrial dysfunction with carcinogenesis led us to investigate mitochondrial changes in p16-deficient cells. We found that compared to wild-type (WT) primary mouse fibroblasts (PMFs), p16-deficient cells exhibited significantly greater mitochondrial mass as reflected by staining with MitoTracker^®^ Green (Figure 1a), which accumulates in mitochondria. The higher MitoTracker fluorescence was consistently observed in several different preparations of p16^−/−^ PMFs from different mice. Elevated MitoTracker staining was consistent with increased expression of representative outer and inner membrane and matrix proteins compared to WT PMFs (Figure 1b). These include subunit proteins involved in different respiratory complexes, and include both nuclear DNA-encoded (SDHA, UQCRC2, ATP5A) and mitochondrial DNA-encoded (ND4) mitochondrial respiration-associated proteins. The non-respiratory-associated outer mitochondrial membrane voltage-dependent anion channel (VDAC) protein was also more highly expressed in p16^−/−^ PMFs (Figure 1b).

Given the increased mitochondrial mass associated with p16-deficiency, we assessed the relative expression of several proteins known to regulate mitochondrial biogenesis, namely the PGC-1 coactivators [13]. Levels of PRC (PGC-1-related coactivator) [20] were increased in p16^−/−^ compared to WT PMFs, while PGC1α was not (Figure 1c). We were unable to detect PGC1-β in either p16^−/−^ or WT PMFs (not shown). Furthermore, expression of TFAM (transcription factor A, mitochondrial) [21], which is usually regulated by PRC [22], was also increased in p16^−/−^ compared to WT PMFs (Figure 1c). Upregulation of both PRC, which resides in the nucleus [13], and TFAM, which resides in the mitochondria and directly controls replication and transcription of the mitochondrial genome [13], suggest a coordinated transcriptional response causing enhanced mitochondrial biogenesis in p16-deficient cells.

### Loss of p16 results in decreased respiratory capacity and altered mitochondrial morphology and membrane potential

**Figure 2:**
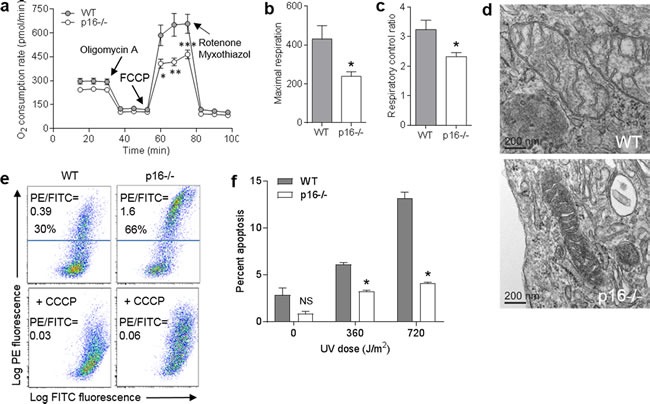
Loss of p16 results in decreased respiration capacity and mitochondrial changes **a.** Oxygen consumption rate measured in wild-type (WT) and p16^−/−^ primary fibroblasts with timed addition of oligomycin A (1 μg/ml), FCCP (0.5 μM), and a mixture of rotenone (1 μM) and myxothiazol (1 μM). Error bars indicate SEM from 9 determinations. *P = .02, **P = .007, ***P = .01. **b.** Maximal respiration. Values calculated from data in **a.**. *P = .02. **c.** Respiratory control ratio. Values calculated from data in **a.**. *P = .03. **d.** Representative electron micrographs of mitochondria. Scale bar indicates 200 nm. **e.** WT and p16^−/−^ fibroblasts were stained with JC-1, in the absence or presence of 50 μM CCCP. **f.** WT and p16^−/−^ fibroblasts were treated with the indicated doses of UV, then assessed for apoptosis by Annexin V staining 24 h later. Error bars indicate SEM from 3 determinations. *P < .001. NS, not significant.

We next examined whether increased mitochondrial mass in p16^−/−^ cells translated into increased respiratory capacity. We measured oxygen consumption rates in WT and p16^−/−^ PMFs and found that although p16^−/−^ cells express higher levels of respiratory complex proteins (Figure 1b) basal respiration was slightly reduced (time 0, Figure 2a). Moreover, following addition of the uncoupling agent FCCP, the maximal respiration rate was significantly lower in p16^−/−^ compared to WT cells (Figure 2b). The respiratory control ratio, determined from the change in respiration after addition of rotenone and myxothiazol, was also significantly lower in p16^−/−^ *vs*. WT cells (Figure 2c). The reduced maximal respiration and respiratory control ratio indicate an impairment of mitochondrial respiratory function [23], despite enhanced expression of respiratory protein complexes, in p16-deficient cells.

Given this impaired mitochondrial function in p16^−/−^ cells, we next examined mitochondrial morphology and observed marked morphologic differences in the mitochondria between p16^−/−^ and WT cells under transmission electron microscopy. Compared to WT PMFs, mitochondria in p16^−/−^ cells demonstrated much higher electron density (Figure 2d), consistent with the elevated mitochondrial mass (Figure 1a) noted above. While the mitochondrial cristae in WT cells appeared normal, those in p16^−/−^ cells were swollen with elaborated membranes and increased spacing (Figure 2d). Disorganized cristae have been described as an indicator of poor respiratory efficiency [24].

Since many mitochondrial functions involve maintenance and utilization of the mitochondrial membrane potential (∆ψm), we assessed ∆ψm using the potential-sensitive dye JC-1 (red-to-green ratio increases with ∆ψm). We found that ∆ψm was higher in p16-deficient compared to WT PMFs (66% *vs*. 30% red cells, 1.6 *vs*. 0.39 PE to FITC mean fluorescence ratio), and could be reduced to the level of WT cells by addition of the uncoupling agent carbonyl cyanide *m*-chlorophenyl hydrazone (CCCP) (Figure 2e). Given the role of mitochondria in executing cellular apoptosis [25], we asked whether the elevated ∆ψm in p16^−/−^ cells was associated with resistance to apoptosis. As shown in Figure 2f, p16^−/−^ cells were indeed more resistant than WT cells to UV-induced apoptosis.

### p16-deficient cells exhibit higher levels of superoxide and increased motility

**Figure 3:**
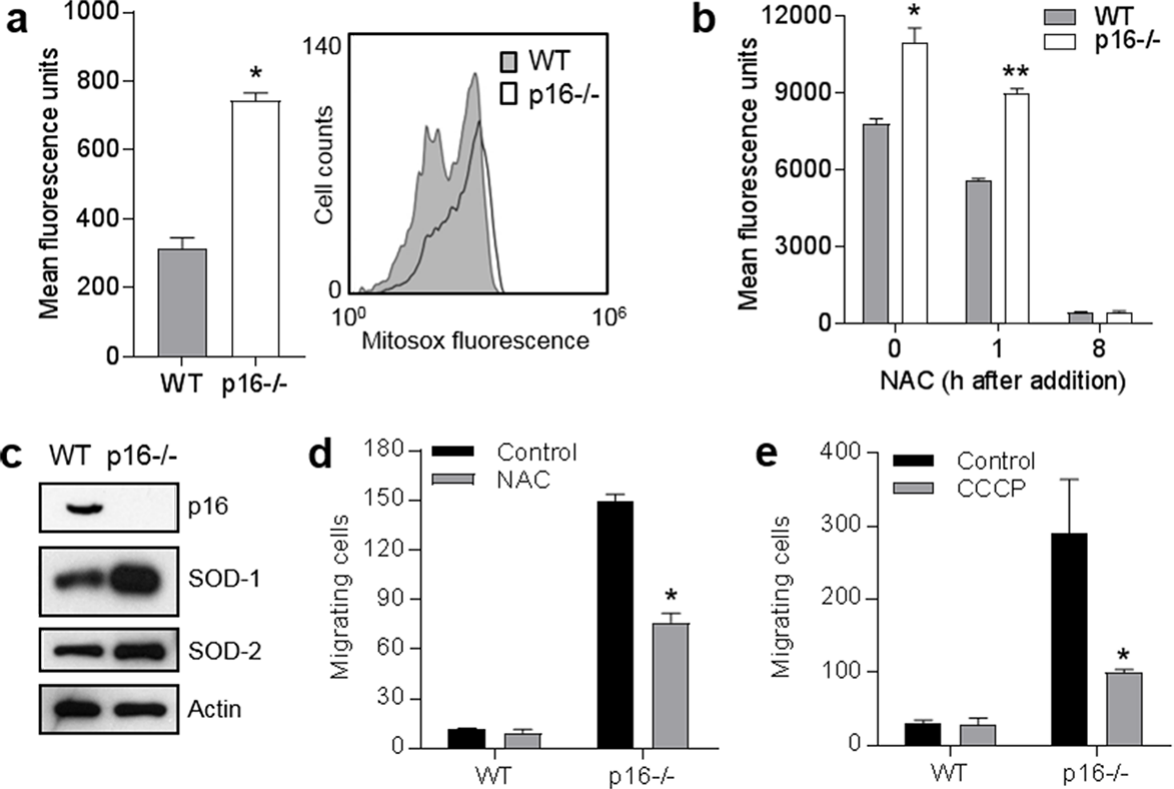
Elevated mitochondrial superoxide in p16-deficient cells is associated with enhanced cell migration **a.** Wild-type (WT) and p16^−/−^ primary fibroblasts were stained with MitoSox-Red and assessed for mitochondrial superoxide by flow cytometry. Error bars indicate SEM from triplicate determinations, *P < .001. Representative histogram at right. **b.** Detection of superoxide in the absence (0 h) or presence of 5 mM N-acetylcysteine (NAC) after 1 or 24 h. Error bars indicate SEM from triplicate determinations, *P < .001, *P = .006. **c.** Western blots of lysates from cells in **a.**. **d.** Wild-type (WT) and p16^−/−^ fibroblasts were subjected to transwell migration assay, in the absence or presence of 5 mM NAC, and migrating cells were counted at 24 h. Error bars indicate SEM from triplicate determinations, *P = .003. **e.** Migration assay as in **d.**, with cells in absence or presence of 50 μM CCCP. Error bars indicate SEM from triplicate determinations, *P = .005.

We had previously shown that loss of p16 resulted in increased levels of intracellular levels of ROS and oxidative DNA damage [7]. Mitochondria are the major source of cellular ROS [26], and it seemed likely that mitochondrial leakage of ROS would result from the unbalanced increase in mitochondrial mass and ∆ψm and impaired respiration we observed in p16^−/−^ cells. Indeed, compared to WT PMFs, p16-deficient cells demonstrated significantly higher levels of mitochondrial superoxide as assessed by MitoSox^®^ Red staining (Figure 3a) that could be normalized by addition of the ROS scavenger N-acetylcysteine (NAC) (Figure 3b). The increased mitochondrial superoxide was not secondary to an impaired antioxidant response, as levels of superoxide dismutase (SOD)-1 and mitochondrial SOD-2 were both upregulated in p16^−/−^ compared to WT PMFs (Figure 3c). Thus, it appears that increased mitochondrial superoxide in the absence of p16 triggers upregulation of SOD proteins to promote detoxification.

Given the known potential role of ROS in promoting cellular migration [17, 19, 27], we assessed the effect of elevated ROS in p16-deficient PMFs on their migratory capacity in a transwell assay. As shown in Figure 3d, migration of p16^−/−^ cells was dramatically enhanced compared to WT cells, and migratory capacity was significantly reduced by addition of NAC in p16^−/−^ but not WT cells. Finally, dissipation of ∆ψm with CCCP significantly reduced the enhanced migration of p16^−/−^ cells, without affecting migration of WT cells (Figure 3e). Importantly, cells were treated with mitomycin C (to block cell division) prior to the assay, to avoid potential artifacts due to differential cell proliferation. Thus, p16 loss is associated with enhanced cellular migration, independent of cell proliferation, and this phenotype is dependent upon elevated ROS and ∆ψm.

### Expression of p16 restores mitochondrial dynamics and motility

**Figure 4:**
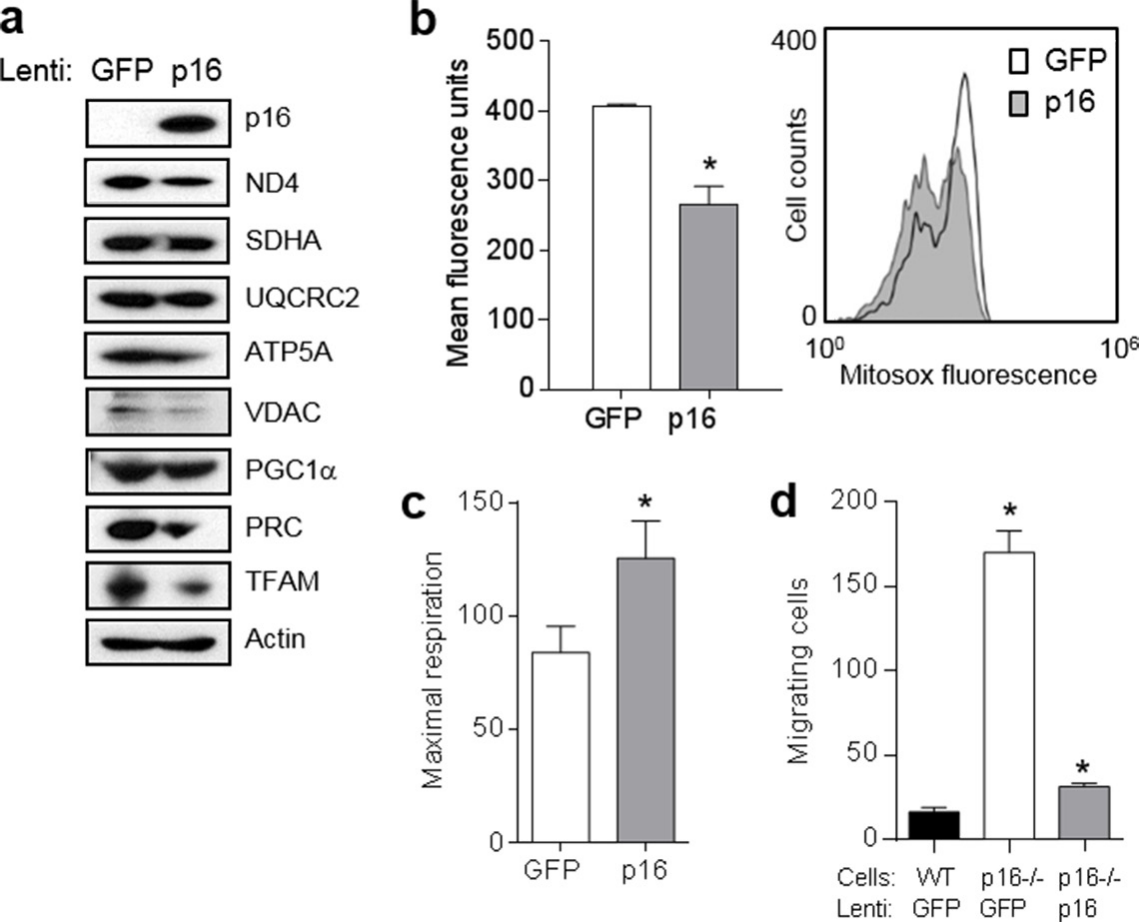
Expression of p16 in p16-deficient fibroblasts restores mitochondrial dynamics and motility **a.** Fibroblasts isolated from p16^−/−^ mice were infected with control (GFP) or p16-expressing lentivirus, then 6 d later lysates were subjected to Western blotting. Representative of two experiments performed. **b.** Cells in **a.** after 6d were analyzed for mitochondrial superoxide by flow cytometry. Error bars indicate SEM from triplicate determinations, *P = .005. Representative histogram at right. **c.** Maximal respiration of cells in **a.**. Error bars indicate SEM from 9 determinations, *P = .05. **d.** Fibroblasts isolated from wild-type (WT) or p16^−/−^ mice were infected with control (GFP) or p16-expressing lentivirus, then 48 h later were placed in migration assay, and migrating cells counted at 24 h. Error bars indicate SEM from triplicate determinations, *P < .001.

To confirm the dependence of this mitochondrial and migratory phenotype on p16, we initially expressed p16 in p16-deficient PMFs using a lentiviral approach. Compared to p16^−/−^ cells infected with a control GFP-expressing lentivirus, similar cells infected with GFP/p16-expressing lentivirus displayed abundant levels of p16 and lower levels of several mitochondrial proteins including ND4, ATP5A and VDAC, and lower levels of the co-activators PRC and TFAM (Figure 4a). Levels of PGC1α were not affected (Figure 4a). The coincident expression of GFP precluded our ability to examine mitochondrial mass using MitroTracker-Green. Mitochondrial superoxide was also significantly reduced by forced p16 expression (Figure 4b). By contrast, mitochondrial respiration was significantly increased by p16 expression (Figure 4c). Finally, expression of p16 reduced the motility of p16^−/−^ cells to that of WT cells (Figure 4d). Thus, forced expression of p16 in p16^−/−^ PMFs was sufficient to reverse all of the mitochondrial and migratory phenotypes observed in p16^−/−^ PMFs.

**Figure 5:**
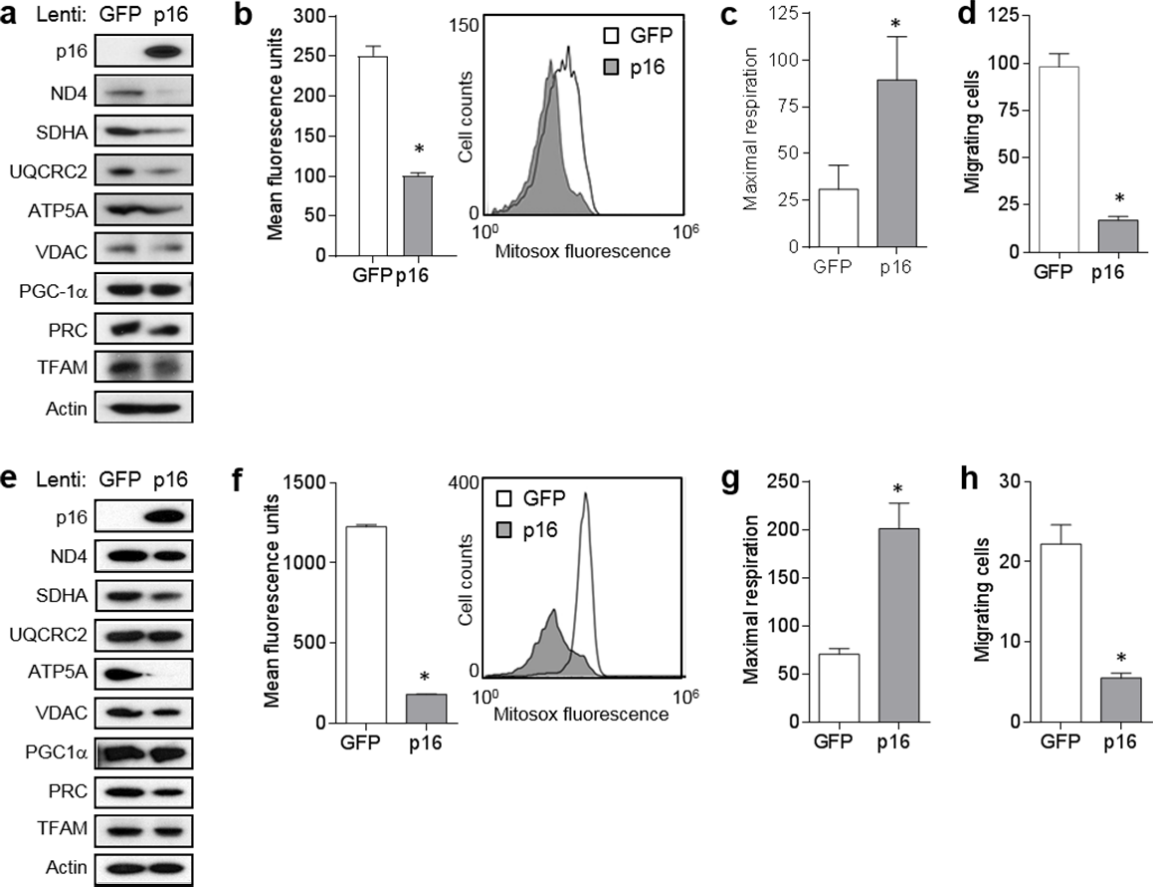
Forced expression of p16 modulates mitochondrial biogenesis, respiration, and motility **a.** A375 human melanoma cells were infected with control (GFP) or GFP/p16-expressing lentivirus, then 6 d later lysates were subjected to Western blotting. **b.** Cells in **a.** analyzed for mitochondrial superoxide by flow cytometry. Error bars indicate SEM from duplicate determinations, *P < .001. **c.** Maximal respiration of cells in **a.**. Error bars indicate SEM from 9 determinations, *P = .04. **d.** A375 cells were infected with control (GFP) or GFP/p16-expressing lentivirus, then 48 h later were placed in migration assay, and migrating cells counted at 24 h. Error bars indicate SEM from triplicate determinations, *P < .001. **e.** Human melanocytes were infected with control (GFP) or GFP/p16-expressing lentivirus, then 6 d later lysates were subjected to Western blotting. **f.** Cells in **e.** analyzed for mitochondrial superoxide by flow cytometry. Error bars indicate SEM from duplicate determinations, *P < .001. **g.** Maximal respiration of cells in **e.**. Error bars indicate SEM from 9 determinations, *P < .001. **h.** Melanocytes were infected with control (GFP) or GFP/p16-expressing lentivirus, then 48 h later were placed in migration assay and migrating cells counted at 48 h. Error bars indicate SEM from triplicate determinations, *P = .003.

Next, we examined whether p16 over-expression in malignant cells and another primary cell type could similarly modulate mitochondrial dynamics and motility. In A375 human melanoma cells (which do not appear to express p16 constitutively), forced expression of p16 resulted in marked downregulation of ND4, SDHA and UQCRC2, while the reduction in PRC and TFAM was less striking (Figure 5a). PGC1α was not affected (Figure 5a). Compared to control GFP-expressing cells, p16-expressing A375 cells displayed significantly lower levels of superoxide (Figure 5b), significantly higher levels of respiration (Figure 5c), and significantly reduced migration (Figure 5d). We similarly used the GFP and GFP/p16 lentiviruses to infect primary human melanocytes, which express very low levels of endogenous p16 [7]. Forced expression of p16 in melanocytes resulted in clearly reduced expression of SDHA, ATP5A and VDAC, while reduction in levels of ND4, PRC and TFAM were more modest (Figure 5e). PGC1α was not affected (Figure 5c). Compared to control cells, over-expression of p16 in melanocytes resulted in significantly lower levels of superoxide (Figure 5f), significantly higher levels of respiration (Figure 5g), and significantly reduced migration (Figure 5h). Thus, over-expression of p16 in both melanoma cells and primary melanocytes yields a reciprocal phenotype to that seen in p16^−/−^ PMFs: reduced mitochondrial protein expression, reduced superoxide, increased respiration, and reduced migration.

### CDK4/6 inhibitors and CDK4 knockdown do not mimic effects of p16 on mitochondrial dynamics and respiration

**Figure 6:**
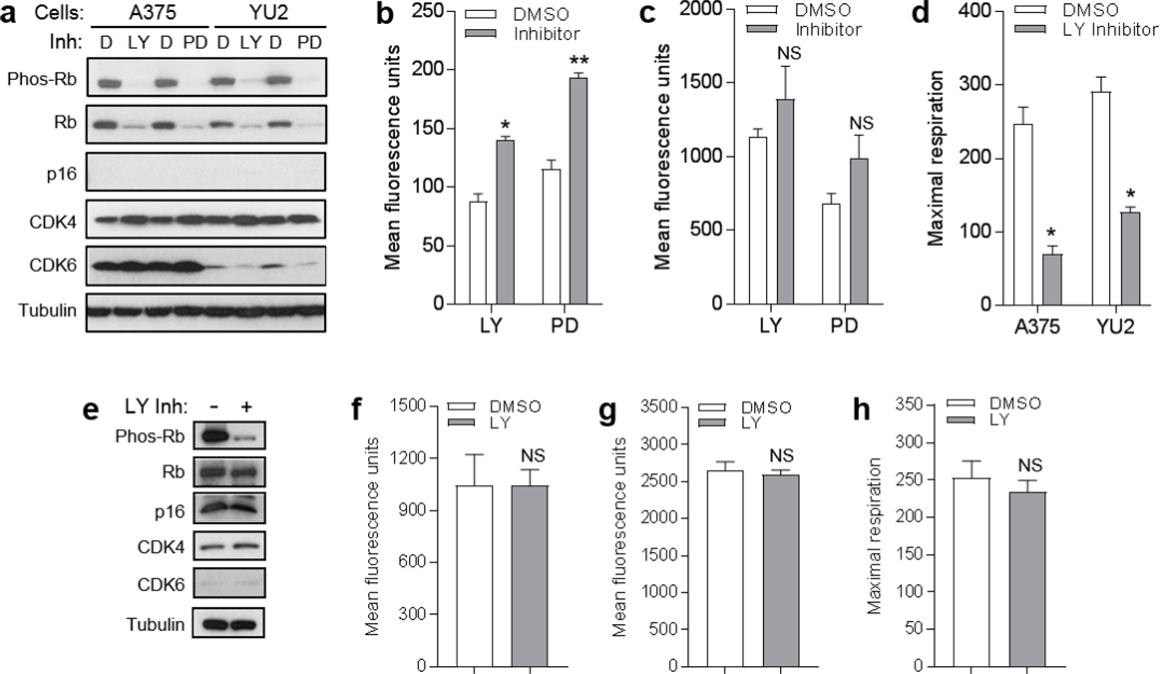
CDK4/6 inhibitors do not mimic effects of p16 on mitochondrial dynamics and respiration **a.** A375 and YU2 melanoma cells were treated with DMSO control **d.**, 1 μM LY-2835219 (LY), or 2 μM PD-0332991 (PD), then lysates were analyzed by Western blotting 24 h later. **b.** A375 cells in **a.** analyzed for mitochondrial superoxide by flow cytometry. Error bars indicate SEM from triplicate determinations; *P = .05, **P < .001. **c.** A375 cells in **a.** analyzed for mitochondrial mass by flow cytometry. Error bars indicate SEM from triplicate determinations; *P = .01. **d.** Maximal respiration of A375 and YU2 cells in **a.**. Error bars indicate SEM from 5 determinations, *P = . < .001. **e.** Human melanocytes were treated with 1 μM LY or DMSO control, then lysates were analyzed by Western blotting 24 h later. **f.** Cells in **e.** analyzed for mitochondrial superoxide by flow cytometry. Error bars indicate SEM from triplicate determinations; NS, not significant. **g.** Cells in **e.** analyzed for mitochondrial mass by flow cytometry. Error bars indicate SEM from triplicate determinations; NS, not significant. **h.** Maximal respiration of cells in **e.**. Error bars indicate SEM from 5 determinations; NS, not significant.

p16 represses cell cycle progression through binding of CDK4/6, which inhibits Rb phosphorylation [2]. We investigated whether p16-mediated modulation of mitochondrial dynamics was operating through this canonical CDK/Rb pathway by testing whether blocking these CDK could mimic the effects observed with p16 over-expression. First, we employed chemical CDK4/6 inhibitors to suppress Rb phosphorylation. As shown in Figure 6a, both LY-2835219 (LY) and PD-0332991 (PD) dramatically reduced levels of phosphorylated Rb in both A375 and YU2 human melanoma cells (which do not express detectable amounts of p16). We noted that these inhibitors also reduce levels of total Rb, which has been recently reported (in bladder cancer cells) to be due to negative feedback of CDK inhibition on *RB* gene transcription [28]. Under these conditions, neither inhibitor affected levels of CDK4 and CDK6 in A375 cells or levels of CDK4 in YU2 cells (Figure 6a). Neither inhibitor caused a reduction in mitochondrial superoxide in A375 cells (Figure 6b), as was seen above with forced p16 expression (Figure 5b). In fact, superoxide levels were increased, perhaps as part of a compensatory response. Similarly, neither inhibitor caused a reduction in mitochondrial mass in A375 cells (Figure 6c). Comparable results were seen in YU2 cells treated with the PD inhibitor (Supplementary Figure 1). In addition, neither inhibitor caused an increase in mitochondrial respiration in either A375 or YU2 cells (Figure 6d), as was seen above with forced p16 expression in A375 cells (Figure 5c). The decreased respiration may represent a compensatory response, as noted above. In primary melanocytes, the LY inhibitor reduced levels of phosphorylated Rb without affecting levels of p16, CDK4, or Rb (Figure 6e). Under these conditions, we did not observe a reduction in mitochondrial superoxide (Figure 6f), as was seen above with forced p16 expression in melanocytes (Figure 5f). Similarly, the LY inhibitor did not cause a reduction in mitochondrial mass in melanocytes (Figure 6g). Finally, the LY inhibitor did not cause an increase in mitochondrial respiration (Figure 6h), as was seen above with forced p16 expression in melanocytes (Figure 5g).

**Figure 7:**
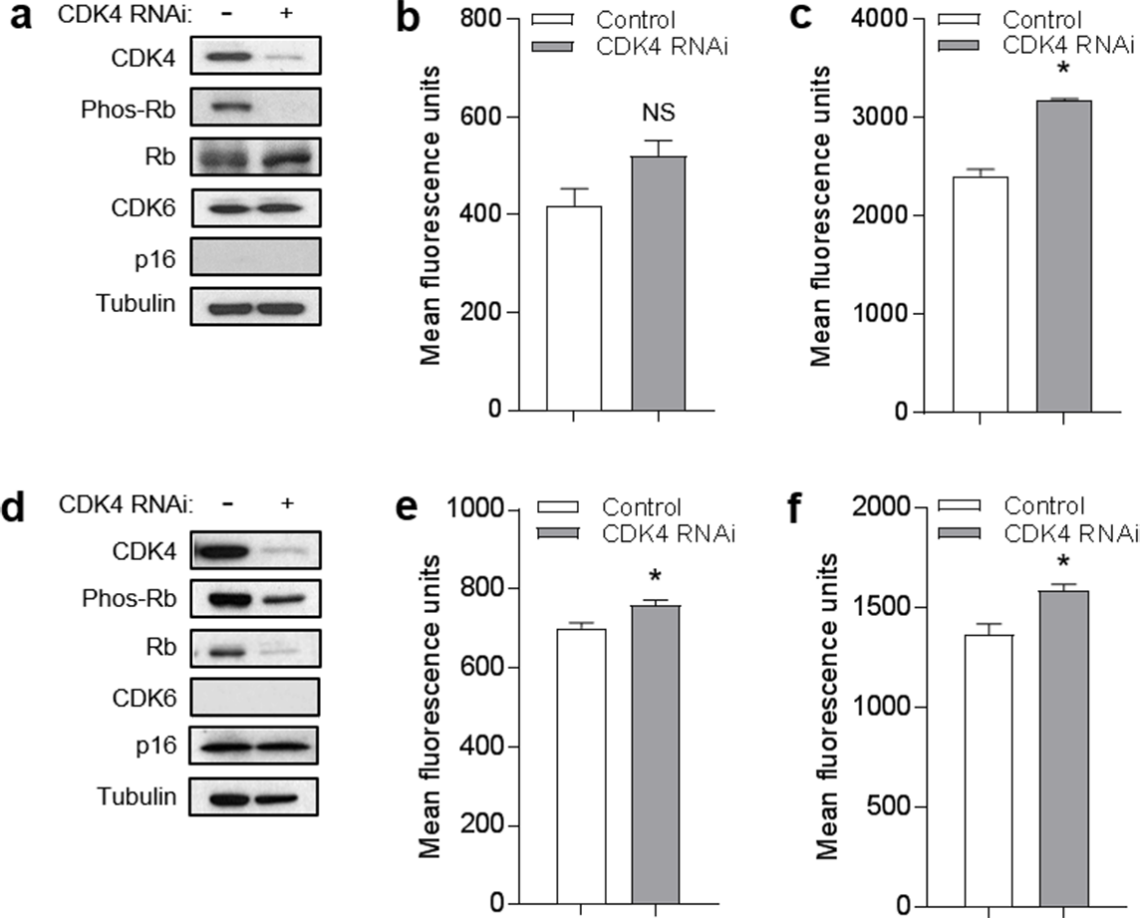
CDK4 knockdown does not mimic effects of p16 on mitochondrial dynamics **a.** YU2 melanoma cells were transfected with control or RNAi specific for CDK4, then lysates were analyzed by Western blotting 72 h later. **b.** Cells in **a.** analyzed for mitochondrial superoxide by flow cytometry. Error bars indicate SEM from triplicate determinations; NS, not significant. **c.** Cells in **a.** analyzed for mitochondrial mass by flow cytometry. Error bars indicate SEM from triplicate determinations; *P < .001. **d.** Human melanocytes were transfected with control or RNAi specific for CDK4, then lysates were analyzed by Western blotting 72 h later. **e.** Cells in **d.** analyzed for mitochondrial superoxide by flow cytometry. Error bars indicate SEM from triplicate determinations; *P = .04. **f.** Cells in **d.** analyzed for mitochondrial mass by flow cytometry. Error bars indicate SEM from triplicate determinations; *P = .04.

As a second approach, we depleted CDK4 using RNAi. Knockdown of CDK4 in YU2 melanoma cells was associated with reduction in phosphorylated Rb without significantly affecting levels of total Rb or CDK6 (Figure 7a). Compared to control cells, YU2 cells with CDK4 depletion did not exhibit a reduction in mitochondrial superoxide (Figure 7b) or mitochondrial mass (Figure 7c). Knockdown of CDK4 in human melanocytes was associated with reduction in phosphorylated Rb without affecting levels of p16 (Figure 7d). Compared to control cells, melanocytes with CDK4 depletion did not exhibit a reduction in mitochondrial superoxide (Figure 7e) or mitochondrial mass (Figure 7f). Thus, neither chemical inhibition of CDK4/6 or knockdown of CDK4, under conditions that caused reduction in phosphorylated Rb, was able to mimic the effects of forced p16 expression on mitochondrial dynamics. These results suggest that mitochondrial regulation by p16 occurs through a CDK4/Rb-independent pathway.

## DISCUSSION

**Figure 8:**
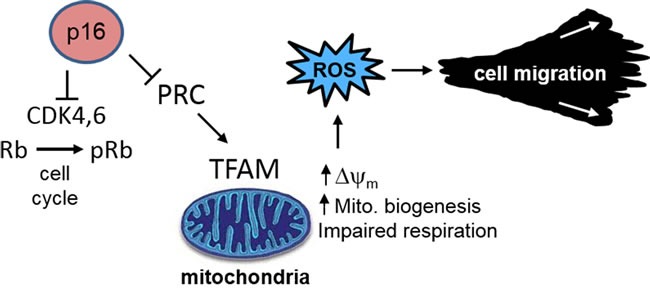
p16 control of mitochondrial biogenesis, ROS, and cell migration Loss of p16 results in aberrant mitochondrial biogenesis, characterized by increased mitochondrial mass but dysfunctional respiration which results in elevated ∆ψm and ROS. These functions are independent of the CDK4/Rb pathway and cell cycle control.

Since the discovery of p16 and its regulatory function in proliferation and transformation over twenty years ago [29, 30], substantial evidence has accumulated to establish its role as a major tumor-suppressor in cancer [31, 32]. Its function in controlling cell cycle progression underlies its additional roles in cellular senescence and aging [33]. As these processes are associated with altered energetic/metabolic needs, these tumor-suppressor functions are likely to be linked to metabolic control mechanisms [34]. For example, the tumor-suppressor p53 is activated by metabolic stress and regulates apoptosis, glycolysis, and mitochondrial respiration through the mTOR and other signaling pathways [35–37]. In addition, other tumor suppressors such as Rb [38] and the E2F transcription factors which are regulated by Rb, have been linked to mitochondrial biogenesis through control of expression of multiple mitochondria-associated genes [39]. We have previously shown that p16 regulates oxidative stress, independent of cell cycle control [7, 8], and it is known that increased ROS can promote carcinogenesis through direct DNA damage [40] and enhancing cell migration [19, 27, 41]. Here we provide initial evidence implicating alterations in mitochondrial biogenesis, structure, and respiratory function in p16-dependent control of ROS and cell migration. We show that p16 is both necessary and sufficient to maintain mitochondrial balance, and in its absence there is an uncoupling of mitochondrial biogenesis and respiration which results in elevated ∆ψm and ROS to promote cellular migration. These functions are independent of the CDK4/Rb pathway and cell cycle control (Figure 8).

The precise mechanisms by which p16 regulates mitochondrial morphology and function remain to be further elucidated. The PGC-1 coactivators (PGC-1α, PGC-1β, PRC) play a central role in mitochondrial biogenesis by coordinately activating multiple genes required for mitochondrial function [13]. Several recent studies have specifically implicated PGC-1α in enhanced mitochondrial function, drug resistance, and tumor cell invasion. Vazquez et al. [11] showed that a subset of melanoma tumors exhibit over-expression of PGC-1α, which mediates increased mitochondrial energy metabolism and ROS detoxification capacity. Knockdown of PGC-1α led to decreased mitochondrial ∆ψm and respiration, increased ROS, and increased susceptibility to ROS-induced apoptosis [11]. LeBleu et al. [12] showed that PGC-1α mediates mitochondrial biogenesis and metastasis of breast cancer cells. By contrast, we did not find that increased mitochondrial biogenesis in p16-deficient cells was associated with altered expression of PGC-1α. Rather, we observed upregulated expression of PRC and TFAM (a known target of PRC activation) [22]. Overexpression of mitochondrial respiratory proteins and increased mitochondrial mass, however, did not improve respiratory function or efficiency in p16-deficient cells and was associated with morphological alterations (particularly in the cristae). Coordinate expression of mitochondrial proteins and organized cristae determine mitochondrial respiratory efficiency [24]. It is likely that PRC/TFAM and the downstream respiratory proteins are upregulated as part of a compensatory response, but it is not clear why this response is ineffective in restoring efficient respiratory metabolism.

It appears that the mitochondria are effective at the initial stages of the electron transport chain to establish a robust mitochondrial membrane potential. There appears to be a defect in the reduction of oxygen to water catalyzed by cytochrome c oxidase (Complex IV), however, as indicated by impaired maximal respiration. It is also possible that there is a defect in the function of ATP synthase (Complex V), which consumes the membrane potential to generate ATP, based on the consistently elevated potential. Similarly, Kim et al. [42] found that deficiency of the cyclin-dependent kinase inhibitor p21 led to increases in mitochondrial mass and expression of coactivators, but respiratory function was impaired. Bonawitz et al. [43] reported that yeast mutants of mitochondrial RNA polymerase also displayed mitochondrial imbalance characterized by increased ROS production and depressed respiration. Finally, our findings are consistent with those of Capparelli et al [44], who reported that p16 over-expression in cancer-associated fibroblasts actively suppresses the expression of OXPHOS subunits, especially related to Complex I, III, IV and V.

The role of Rb in mitochondrial regulation has been studied primarily in the setting of *RB* gene deficiency. Sankaran et al. [45] found that Rb-null mouse erythroid progenitors fail to activate the mitochondrial biogenesis program required for differentiation. Similarly, Ciavarra and Zacksenhaus [46] reported that Rb-deficient mouse muscle cells fail to develop due to mitochondrial loss. In prior work [7], we showed that RNAi-mediated depletion of Rb in human melanocytes failed to recapitulate the effects of p16 loss on ROS accumulation but did not examine mitochondrial biogenesis. The relationship between CDK4 and mitochondrial function has primarily been studied in Drosophila. Frei, Edgar and colleagues [47–49] reported that expression of cyclin D-CDK4 increased mitochondrial biogenesis while flies deficient in CDK4 exhibited defects in mitochondrial biogenesis. A connection between CDK4 and mitochondrial biogenesis is consistent with our findings of activation of mitochondrial biogenesis with p16 loss and its suppression with p16 over-expression. However, our demonstration that neither chemical inhibition of CDK4/6 nor CDK4 knockdown (under conditions that blocked Rb phosphorylation) could mimic the effects of p16 loss suggests that mitochondrial control by p16 is mediated along a CDK/Rb-independent pathway.

Increased mitochondrial biogenesis and respiratory capacity can support increased proliferation and metabolic activity associated with cancer growth, and promote cellular motility through increased generation of ROS [10, 50–55]. Ishikawa et al. [56] showed that particular mutations in mitochondrial DNA increased ROS generation which could regulate tumor cell metastasis. ROS may promote cellular migration through a variety of pathways including caveolin-1 [41], HIF1-α [19], NFκβ [27], and Rho/Rac kinases [57, 58]. Presumably the increased migration we observed in p16-deficient cells is a consequence of increased ROS, but we have not examined the potential role of any of these pathways. The fact that addition of NAC or CCCP to p16^−/−^ cells did not completely normalize migration (to the level seen in WT cells), suggests that other factors may indeed be involved. Al-Ansari et al. [59] showed that p16 suppresses migration of breast stromal fibroblasts, and in this system the Akt pathway was involved. Our results are also consistent with a previous report by Pelicano et al. [17], who reported that increased ROS may also be associated with mitochondrial dysfunction, and promote motility in this context. In addition to endogenous effects on tumor cells, mitochondrial dysfunction in the stroma may promote migration of adjacent tumor cells through ROS production and paracrine cytokine effects. Taddei et al. [18] showed that conditioned media from fibroblasts with mitochondrial dysfunction could stimulate melanoma cell invasion, but they did not identify the cytokine(s) involved. Finally, it is important to note that ROS and mTOR mutually stimulate each other and mTOR can be inhibited by NAC [60].

The second protein encoded by *CDKN2A* is Arf (p14) [2], which similarly may regulate cell cycle or senescence in response to DNA damage and other signals [61], and has also been identified as a regulator of mitochondrial function. Reef et al. [62] found that Arf can localize to mitochondria to regulate autophagy and apoptosis. More recently, Christensen et al. [63] reported that Arf controls superoxide production by translocating to mitochondria in pre-senescent cells and binding to Bcl-xL. Arf-deficient cells also demonstrated enhanced respiratory capacity, but since the cells used in these experiments were also deficient in p16 it is not clear to what extent the effects on mitochondrial function could be attributed to Arf *vs*. p16 [63]. By contrast, we have not found p16 to be localized in the mitochondria in unstressed fibroblasts (B. Liu and Grossman, unpublished observations), and noted decreased mitochondrial respiration and abnormal mitochondrial structure in the cells used here which were deficient in p16 but not Arf. These two tumor suppressors have been most studied in the context of melanoma, likely because germline *CDKN2A* mutations in some families confer increased melanoma risk [4, 5]. In human melanoma, p16 may be a more important tumor suppressor than Arf given that mutations (in exon 1β of *CDKN2A*) which exclusively affect Arf are generally not seen in melanoma tumors [64].

Interestingly, there is a recent report [65] that mitochondrial DNA is increased in peripheral blood of carriers of specific p16 mutations that we have identified as defective in oxidative regulation [8].

In summary, we have shown that the increased ROS we previously reported in p16-deficient cells [7] is a consequence of mitochondrial imbalance characterized by increased mitochondrial mass and impaired respiration, leading to elevated mitochondrial ∆ψm and ROS (Figure 8). These findings represent a new, unforeseen function of p16 which may underlie critical aspects of its tumor suppressive function. It is likely that these mitochondrial consequences of p16 loss, in addition to enhanced proliferation and impaired senescence, underlie the selection of cells with loss of p16 function in developing melanoma and other tumors.

## MATERIALS AND METHODS

### Cell culture

PMFs were isolated from newborn wild-type mouse (FVB) and background-matched p16^−/−^Arf^+/+^ (#01XE4, FVB.129-*Cdkn2a*^tm2.1Rdp^) homozygous mice [66], obtained from the National Cancer Institute (Rockville, MD) as described previously [7]. All procedures were approved by Institutional Animal Care and Use Committee of the University of Utah. Freshly isolated PMFs were passaged once and then aliquotted and stored under liquid nitrogen. Cells were thawed, expanded in DMEM medium containing 10% FCS, and used over a period of several weeks. Human melanoma cells lines A375 (American Type Culture Collection, Manassas, VA) and YU2 (YUSAC2) were maintained in DMEM containing 5% FCS as previously described [67]. Human melanocytes were isolated from neonatal foreskins and maintained as described previously [68]. For forced expression of p16, cells were infected with GFP- or GFP/p16-expressing lentiviruses as previously described [7].

### Mitochondrial mass and superoxide

Mitochondrial mass was measured using MitoTracker^®^ Green FM (Life Technologies, Carlsbad, CA), and mitochondrial superoxide was measured using MitoSox^®^ Red (Life Technologies), according to the manufacturer's instructions. Briefly, cells were trypsinized, washed, then incubated with 200 nM MitoTracker or 5 μM for MitoSox for 15 min, then run on a FACScan flow cytometer (BD Biosciences, San Jose, CA). In some experiments, cells were cultured with freshly prepared NAC (Sigma Chemical Co., St. Louis, MO) at a final concentration of 5 mM.

### Western blotting

Immunoblotting was performed as described previously [7]. The following specific primary antibodies were used against p16 (sc-74400, 1:200; sc-1661, 1:500), SOD1 (sc-11407, 1:500), SOD2 (sc-30080, 1:200), ND4 (sc-20499-R, 1:200), PGC-1α (sc-13067, 1:200), PGC-1β (sc-373771, 1:200), PRC (sc-135516, 1:200), TFAM (sc-166965, 1:200), and Rb (sc-102, 1:500) obtained from Santa Cruz Biotechnology (Santa Cruz, CA). When the p16 antibodies were no longer available from Santa Cruz, we used a different antibody (NA29, 1:100) from EMD Millipore (Billerica, MA). The β-actin antibody was used at a 1:1000 dilution and obtained from Sigma-Aldrich (A-3853, St. Louis, MO, USA). The ATP5A (ab14746, 1;200), UQCRC2 (ab14745, 1:200), and tubulin (ab21058, 1:5000) antibodies were obtained from Abcam (Cambridge, MA). The SDHA antibody (MS 204M) was used at a 1:200 dilution and obtained from Mitoscience (Eugene, OR). The VDAC antibody (PAI-954A) was used at a 1:200 dilution and obtained from ABR Affinity Bioreagents (Golden, CO). The phospho-Rb (8180S, 1:1000), CDK4 (12790S, 1:1000), and CDK6 (3136S, 1:500) antibodies were obtained from Cell Signaling Technology (Beverly, MA).

### Mitochondrial respiration

Oxygen consumption (respiration) measurements were determined using a Seahorse XF24 analyzer (Seahorse Bioscience, North Billerica, MA). Cells (3×10^4^ ) were seeded in XF24 culture plate wells in DMEM growth medium with the first row as empty control, and remaining wells for replicates. After overnight growth, oxygen consumption rate was measured by automated sequential additions of drugs with these final concentrations: oligomycin A (1 μg/mL), carbonyl cyanide-p-trifluoromethoxyphenylhydrazone (FCCP, 0.5 μM), and a mixture of rotenone (1 μM) and myxothiazol antimycin A (1 μM). The respiratory control ratio was determined by the oxygen consumption during non-mitochondrial respiration (after addition of rotenone and myxothiozol) divided by that during proton leak.

### Transmission electron microscopy

Sample preparation and image acquisition was performed by the Electron Microscopy Core at the University of Utah Health Sciences Center. Briefly, cells were grown in a 75-cm^2^ cell culture flask to sub-confluency. Cells were rinsed once with warm PBS and then fixed by adding 4 mL 2.5% glutaraldehyde in 0.1 M sodium cacodylate (pH 7.4). Cells were scraped from the flask, transferred to a 15 ml tube, and then stored in fixative at 4°C overnight. After fixing, washing, dehydrating, infiltration, and embedding, 100 nm sections were cut and stained with uranyl acetate and lead citrate. Images were obtained with a JEOL JEM-1400 Plus transmission electron microscope.

### Mitochondrial membrane potential

∆ψm was measured using the potential-sensitive dye JC-1 from Life Technologies (M34152), according to the manufacturer's instructions. The dye was added to the cell culture medium at a final concentration of 2 μM, then 30 min later cells were trypsinized and analyzed by flow cytometry. In some cases, the uncoupling agent CCCP (Life Technologies) was added at a final concentration of 50 μM to the growth medium before adding the JC-1 dye.

### UV-induced apoptosis

Cells were irradiated using FS20T12-UVB bulbs (National Biological Corp., Twinsburg, OH) filtered by a Kodacel TA422 membrane as described previously [69]. Apoptosis was detected using an Annexin V-FITC Apoptosis Detection Kit (BD Biosciences, San Diego, CA) following the manufacturer's instructions.

### Cell migration

Transwell migration assays were performed as previously described [67]. Briefly, 5×10^3^ cells were loaded onto fibronectin-coated inserts and incubated for 24 h. Migrating cells on the lower membrane surface were stained with DAPI and visualized by fluorescence microscopy.

### CDK inhibition and knockdown

CDK4/6 inhibitors PD0332991 (S1116) and LY2835219 (S7158) were obtained from Selleckchem (Houston, TX) and stock solutions prepared in dimethylsulfoxide. They were added to cells 24 h prior to Western blotting or respiration analyses. For CDK4 knockdown, cells were transfected with siRNA targeting human CDK4 (Ambion #4390824, s2824) or a non-silencing control (Ambion #4390843) and Lipofectamine RNAiMax Transfection Reagent (Invitrogen #13778150) obtained from ThermoFisher Scientific (Waltham, MA) according to the manufacturer's protocol. Briefly, the cells were plated at about 60-80% confluency in a six well plate. The next day, the media was changed to Opti-MEM medium (ThermoFisher Scientific). The respective siRNAs (60pmole) were diluted to 150 μL in Opti-Mem and then separately mixed 1:1 with Transfection Reagent (9 μL diluted to 150 μL in Opti-Mem) and then incubated for 5 min at room temperature. The siRNA-lipid complexes were then added to the cells and after 6-8 hours of incubation, equal volumes of DMEM containing 10% FCS were added and cells harvested 72 h later.

### Statistical analysis

Statistical analyses were performed using Prism software (GraphPad, La Jolla, CA). P-values ≤ 0.05 were considered statistically significant.

## SUPPLEMENTARY MATERIALS FIGURE



## Abbreviations

CDK: cyclin-dependent kinase
EM: electron microscopy
PGC-1: peroxisome proliferator-activated receptor γ coactivator
PMF: primary mouse fibroblasts
PRC: PGC-1-related coactivator
Rb: retinoblastoma protein
ROS: reactive oxygen species
TFAM: transcription factor A mitochondrial
VDAC: voltage-dependent anion channel
