# Supplementary material for: Loss of p16INK4A stimulates aberrant mitochondrial biogenesis through a CDK4/Rb-independent pathway
Source: Oncotarget. 2017 Aug 3;8(34):55848–62. doi: 10.18632/oncotarget.19862 (PMC5593528; doi:10.18632/oncotarget.19862)
Supplement: Supplementary file 1 [file oncotarget-08-55848-s001.pdf]

Loss of p16^{INK4A} stimulates aberrant mitochondrial biogenesis through a CDK4/Rb-independent pathway

SUPPLEMENTARY MATERIALS

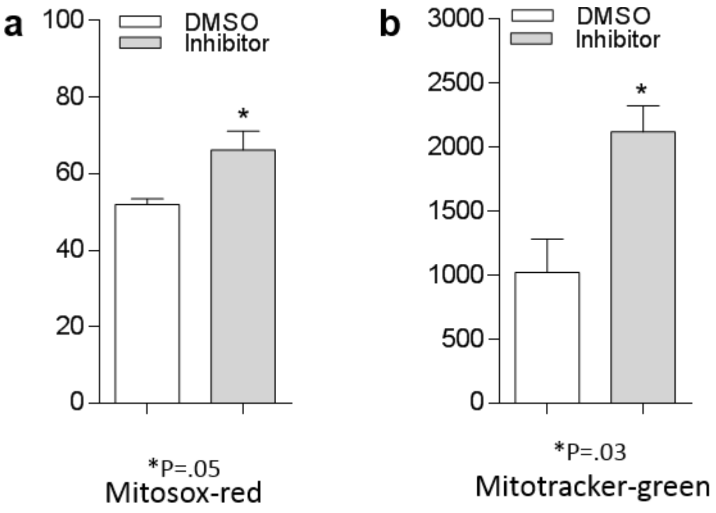

Suppl Fig. 1 CDK4/6 inhibitors do not mimic effects of p16 on mitochondrial dynamics. (**a**) YU2 melanoma cells were treated with DMSO control or 2 μM PD-0332991 (Inhibitor), then 24 h later cells were analyzed for mitochondrial superoxide by flow cytometry using Mitosox-red. Error bars indicate SEM from triplicate determinations; *P=.05. (**b**) Cells in (a) analyzed for mitochondrial mass by flow cytometry using Mitotracker-green. Error bars indicate SEM from triplicate determinations; *P=.03.
